# RbH_2_AsO_4_

**DOI:** 10.1107/S1600536813026676

**Published:** 2013-10-02

**Authors:** Berthold Stöger

**Affiliations:** a Institute for Chemical Technologies and Analytics, Division of Structural Chemistry, Vienna University of Technology, Getreidemarkt 9/164-SC, A-1060 Vienna, Austria

## Abstract

RbH_2_AsO_4_, rubidium di­hydrogenarsenate (RDA), was synthesized by partial neutralization of an aqueous H_3_AsO_4_ solution with aqueous Rb_2_CO_3_. Its paraelectric room-temperature phase is composed of virtually regular tetra­hedral [AsO_2_(OH)_2_]^−^ anions and Rb^+^ cations, both located on -4 positions. The [AsO_2_(OH)_2_] units are connected *via* O—H⋯O hydrogen bonds into a three-dimensional network, whereby the H atoms are equally disordered between the O atoms. The Rb^+^ cations are located in channels running along the <100> directions and coordinated by eight O atoms located at the vertices of a snub disphenoid.

## Related literature

For isotypic phases, see: Al-Karaghouli *et al.* (1978 ▶); Delain (1958 ▶); Ferrari *et al.* (1956 ▶); Helmholtz & Levine (1942 ▶); Novotny & Szekely (1952 ▶); West (1930 ▶); Tenzer *et al.* (1958 ▶). For related phases, see: Stöger *et al.* (2012 ▶). For isoformular phases crystallizing in a different structure type, *viz.*LiH_2_PO_4_, see: Catti & Ivaldi (1977 ▶); Catti & Ferraris (1974 ▶); Nelmes & Choudhary (1978 ▶); Fanchon *et al.* (1987 ▶). For phase transition, see: Fairall & Reese (1974 ▶). For physical properties of RDA and isotypic analogs, see: Ichikawa *et al.* (2001 ▶); Shen (1984 ▶); Negres *et al.* (2005 ▶). For crystal growth, see: Rashkovich (1991 ▶). For bond-valence analyses, see: Brown & Altermatt (1985 ▶). The extinction correction is described by Becker & Coppens (1974 ▶).

## Experimental

### 

#### Crystal data

RbH_2_AsO_4_
*M* *_r_* = 226.4
Tetragonal, 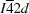

*a* = 7.7865 (9) Å
*c* = 7.466 (2) Å
*V* = 452.64 (14) Å^3^
*Z* = 4
Mo *K*α radiation
μ = 18.07 mm^−1^
*T* = 295 K
0.50 × 0.29 × 0.27 mm

#### Data collection

Bruker Kappa APEXII CCD diffractometer
Absorption correction: multi-scan (*SADABS*; Bruker, 2013 ▶) *T*_min_ = 0.004, *T*_max_ = 0.009
9202 measured reflections
955 independent reflections
567 reflections with *I* > 3σ(*I*)
*R*_int_ = 0.076

#### Refinement

*R*[*F* ^2^ > 2σ(*F* ^2^)] = 0.025
*wR*(*F* ^2^) = 0.032
*S* = 1.25
955 reflections
20 parameters
1 restraint
All H-atom parameters refined
Δρ_max_ = 1.02 e Å^−3^
Δρ_min_ = −0.53 e Å^−3^
Absolute structure: Flack (1983) ▶, 409 Friedel pairs
Absolute structure parameter: −0.010 (13)

### 

Data collection: *APEX2* (Bruker, 2013 ▶); cell refinement: *SAINT-Plus* (Bruker, 2013 ▶); data reduction: *SAINT-Plus*; program(s) used to refine structure: *JANA2006* (Petříček *et al.*, 2006 ▶); molecular graphics: *ATOMS* (Dowty, 2006 ▶); software used to prepare material for publication: *publCIF* (Westrip, 2010 ▶).

## Supplementary Material

Crystal structure: contains datablock(s) global, I. DOI: 10.1107/S1600536813026676/pk2499sup1.cif

Structure factors: contains datablock(s) I. DOI: 10.1107/S1600536813026676/pk2499Isup2.hkl

Supplementary material file. DOI: 10.1107/S1600536813026676/pk2499Isup3.cml

Additional supplementary materials:  crystallographic information; 3D view; checkCIF report

## Figures and Tables

**Table:** 

Rb—O	3.0890 (17)
Rb—O^i^	2.9304 (12)
As—O	1.6828 (11)

**Table:** 

O—As—O^ii^	109.80 (5)
O—As—O^iii^	109.31 (5)

Symmetry codes: (i) 
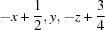
; (ii) 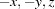; (iii) 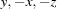.

**Fig. 1.**
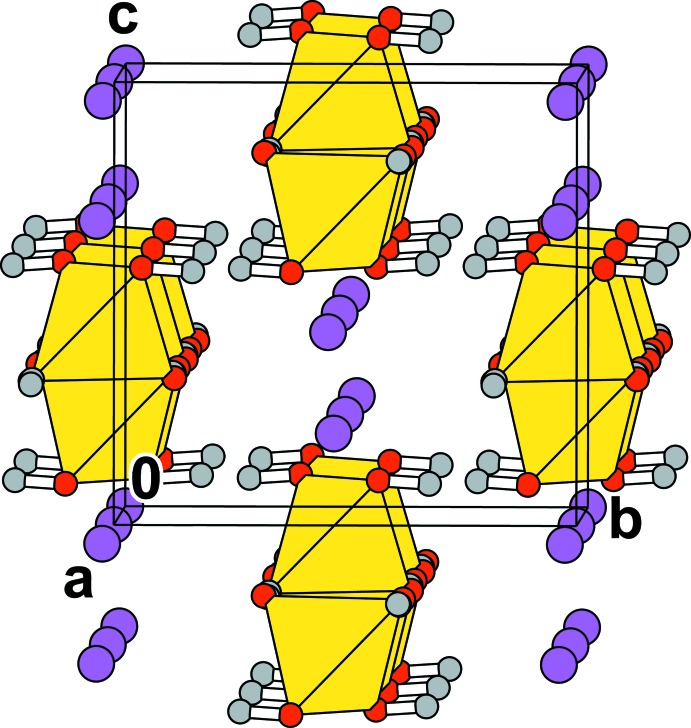
The crystal structure of the paraelectric room temperature phase of RDA viewed approximately down [100]. [AsO4] tetrahedra are drawn in yellow; Rb, O and H atoms are represented by purple, red and white spheres of arbitrary radii.

**Fig. 2.**
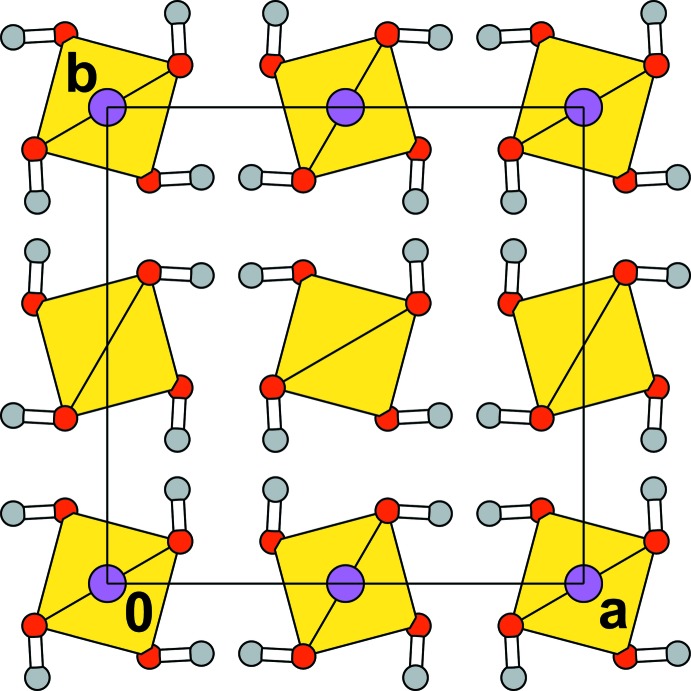
Crystal structure of the paraelectric room temperature phase of RDA viewed down the tetragonal axis [001]. Atom colour codes as in Fig. 1.

**Fig. 3.**
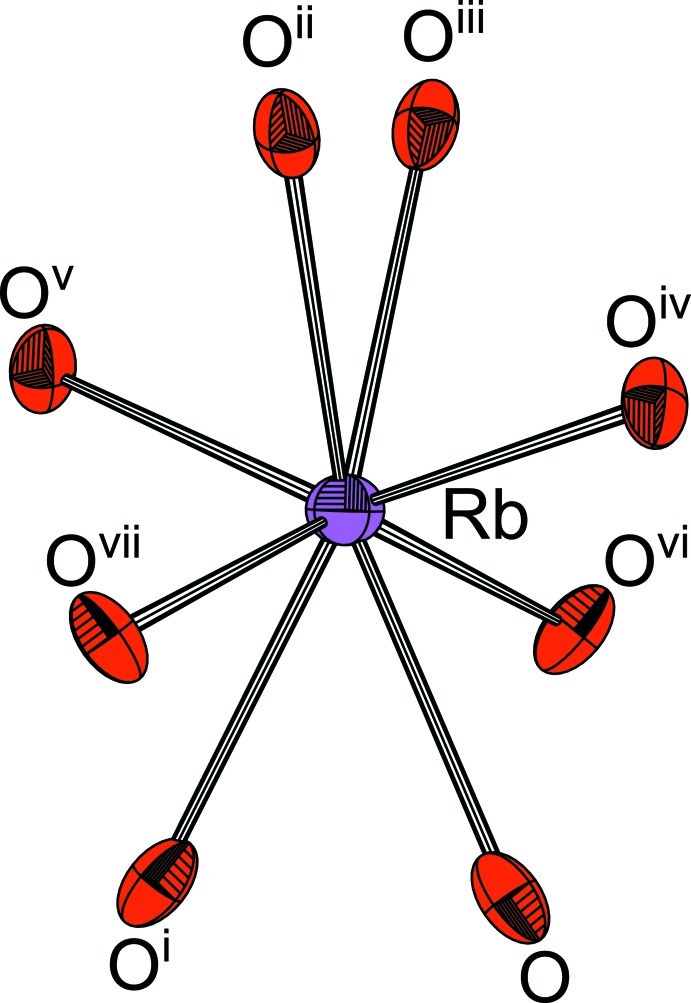
Coordination polyhedron of Rb in the paraelectric room temperature phase of RDA. Rb and O are represented by purple and red ellipsoids drawn at 75% probability level. Symmetry codes: (i) -x, -y, z; (ii) y, -x, -z + 1; (iii) -y, x, -z + 1; (iv) -x + 1/2, y, -z + 3/4; (v) x - 1/2, -y, -z + 3/4; (vi) -y, -x + 1/2, z + 1/4; (vii) y, x - 1/2, z + 1/4

## supplementary crystallographic information

### 1. Comment

During formation studies and subsequent structure analysis of compounds in the system K_2_O–As_2_O_5_–H_2_O (Stöger *et al.*, 2012), related alkali phosphates and arsenates with formula type *M*H_2_*X*O_4_ (*M* = K, Rb, Cs, NH_4_; *X* = P, As) came into attention. With the exception of CsH_2_PO_4_, dihydrogenphosphates and -arsenates *M*H_2_*X*O_4_ (*M* = K, Rb, Cs, NH_4_; *X* = P, As) are isotypic at room temperature and said to belong to the KH_2_PO_4_ (KDP) family. Members of the KDP family are ferroelectrics with low *T_c_* (Ichikawa *et al.*, 2001) and feature non-linear optical (NLO) properties (Shen, 1984). They have been intensely studied for their physical properties paired with a simple crystal-chemistry. Moreover, they are of technical importance in optical applications due to their favourable transparency, high damage threshold (Negres *et al.*, 2005) and ready access to large single crystals (Rashkovich, 1991). Notably, KDP is used as a standard NLO active compound to evaluate the performance of novel NLO materials.

Structural data was published for all members of the KDP family with the exception of RbH_2_AsO_4_ (RDA): KH_2_PO_4_ (West, 1930), RbH_2_PO_4_ (Al-Karaghouli *et al.*, 1978), (NH_4_)H_2_PO_4_ (Tenzer *et al.*, 1958), KH_2_AsO_4_ (Helmholtz & Levine, 1942), CsH_2_AsO_4_ (Ferrari *et al.*, 1956) and (NH_4_)H_2_AsO_4_ (Delain, 1958). The germanate SrH_2_GeO_4_ (Novotny & Szekely, 1952) crystallizes likewise in the KDP structure type. The dihydrogenphosphates and arsenates with larger or smaller alkali metals crystallize in different structure types: LiH_2_PO_4_ (Catti & Ivaldi, 1977), NaH_2_PO_4_ (Catti & Ferraris, 1974), CsH_2_PO_4_ (Nelmes & Choudhary, 1978) and LiH_2_AsO_4_ (Fanchon *et al.*, 1987).

At room temperature RDA, like all members of the KDP family, exists in the tetragonal paraelectric phase. Below *T_c_* = 110 K it transforms into the orthogonal ferroelectric phase (Fairall & Reese, 1974). The room temperature phase of RDA crystallizes in *I*42*d* symmetry. The crystal structure is made up of one [AsO_2_(OH_2_)]^-^ anion and one Rb^+^ cation, both located on 4 positions. The [AsO_2_(OH_2_)] tetrahedra are virtually regular (As—O bond lengths 1.6828 (11) Å; O—As—O angles 109.80 (5)° and 109.31 (5)°). They are connected *via* hydrogen bonding in the <100> directions, forming a three dimensional network (Figs. 1 and 2). Thus, every O atom is either donor or acceptor of an O—H···O hydrogen bond, whereby the proton is equally disordered between both oxygen atoms.

The total bond valence sum (BVS) of the unique O atom calculated using Σexp((*r*_0_-*r*)/*b*) and the parameters of Brown and Altermatt (1985) for Rb^I^—O (r_0_=2.263 Å, *b*=0.37) and As^V^—O (r_0_=1.767 Å, *b*=0.37) is 1.527 (4) valence units (v.u.). This value is in good agreement with the observed disorder, as it lies halfway between the ideal values of O^2-^ and O^-^ (2 and 1 v.u., respectively).

The Rb^+^ cation is located in channels running along the <100> directions (Fig. 1). It is coordinated by eight O atoms located at the vertices of a snub disphenoid (Fig. 3). The total BVS of Rb^+^ calculates as 1.0878 (15) v.u. using the parameters above, which is in excellent agreement with the expected value (1 v.u.). More remote O atoms are located at 4.3005 (12) Å from the Rb^+^ ion and can therefore not be considered part of the coordination sphere (contribution of 0.004 v.u.).

### 2. Experimental

Rb_2_CO_3_ and H_3_AsO_4_ were obtained commercially and used without purification. 1 g 80% *aq*. H_3_AsO_4_ was dissolved in 10 ml water and titrated against *aq*. Rb_2_CO_3_ using one drop of methyl red in EtOH as indicator. The water was evaporated and the residue recrystallized from a small amount of water and washed with acetone to obtain large single crystals of RbH_2_AsO_4_.

### 3. Refinement

An initial model was generated by using the published coordinates of the non-H atoms of the isotypic room temperature phase of RbH_2_PO_4_ (Al-Karaghouli *et al.*, 1978).

The structure was refined against *F* values using the Jana2006 software package (Petříček *et al.*, 2006). The disordered H atom was located in a difference Fourier map and was refined with an occupancy of 0.5. The O—H distance was restrained to 0.850 (1) Å. All non-H atoms were refined with anisotropic displacement parameters.

### Crystal data

**Table:**

RbH_2_AsO_4_	*D*_x_ = 3.321 Mg m^−^^3^
*M**_r_* = 226.4	Mo *K*α radiation, λ = 0.71073 Å
Tetragonal, *I*42*d*	Cell parameters from 2250 reflections
Hall symbol: I -4 2bw	θ = 3.7–44.1°
*a* = 7.7865 (9) Å	µ = 18.07 mm^−^^1^
*c* = 7.466 (2) Å	*T* = 295 K
*V* = 452.64 (14) Å^3^	Block, clear colourless
*Z* = 4	0.50 × 0.29 × 0.27 mm
*F*(000) = 416	

### Data collection

**Table:**

Bruker Kappa APEXII CCD diffractometer	955 independent reflections
Radiation source: X-ray tube	567 reflections with *I* > 3σ(*I*)
Graphite monochromator	*R*_int_ = 0.076
ω and φ scans	θ_max_ = 45.3°, θ_min_ = 3.8°
Absorption correction: multi-scan (*SADABS*; Bruker, 2013)	*h* = −15→15
*T*_min_ = 0.004, *T*_max_ = 0.009	*k* = −15→14
9202 measured reflections	*l* = −14→14

### Refinement

**Table:**

Refinement on *F*	Hydrogen site location: difference Fourier map
Least-squares matrix: full	All H-atom parameters refined
*R*[*F*^2^ > 2σ(*F*^2^)] = 0.025	Weighting scheme based on measured s.u.'s *w* = 1/(σ^2^(*F*) + 0.0001*F*^2^)
*wR*(*F*^2^) = 0.032	(Δ/σ)_max_ = 0.021
*S* = 1.25	Δρ_max_ = 1.02 e Å^−^^3^
955 reflections	Δρ_min_ = −0.53 e Å^−^^3^
20 parameters	Extinction correction: B-C type 1 Gaussian isotropic (Becker & Coppens, 1974)
1 restraint	Extinction coefficient: 4440 (110)
0 constraints	Absolute structure: Flack (1983), 409 Friedel pairs
Primary atom site location: isomorphous structure methods	Absolute structure parameter: −0.010 (13)

### Fractional atomic coordinates and isotropic or equivalent isotropic displacement parameters (Å^2^)

**Table:**

	*x*	*y*	*z*	*U*_iso_*/*U*_eq_	Occ. (<1)
Rb	0	0	0.5	0.01896 (5)	
As	0	0	0	0.01382 (5)	
O	0.15295 (14)	0.08872 (11)	0.12961 (14)	0.0203 (2)	
H	0.147 (6)	0.1975 (7)	0.122 (10)	0.048 (12)*	0.5

### Atomic displacement parameters (Å^2^)

**Table:**

	*U*^11^	*U*^22^	*U*^33^	*U*^12^	*U*^13^	*U*^23^
Rb	0.02023 (8)	0.02023 (8)	0.01640 (11)	0	0	0
As	0.01201 (7)	0.01201 (7)	0.01744 (11)	0	0	0
O	0.0173 (3)	0.0172 (3)	0.0265 (4)	0.0032 (3)	−0.0093 (3)	−0.0063 (4)

### Geometric parameters (Å, º)

**Table:**

Rb—O	3.0890 (17)	Rb—O^vii^	2.9304 (12)
Rb—O^i^	3.0890 (17)	As—O	1.6828 (11)
Rb—O^ii^	3.0890 (17)	As—O^i^	1.6828 (11)
Rb—O^iii^	3.0890 (17)	As—O^viii^	1.6828 (11)
Rb—O^iv^	2.9304 (12)	As—O^ix^	1.6828 (11)
Rb—O^v^	2.9304 (12)	O—H	0.850 (8)
Rb—O^vi^	2.9304 (12)		
			
O—Rb—O^i^	52.94 (3)	O—As—O^i^	109.80 (5)
O—Rb—O^ii^	143.26 (3)	O—As—O^viii^	109.31 (5)
O—Rb—O^iii^	143.26 (3)	O—As—O^ix^	109.31 (5)
O—Rb—O^iv^	82.32 (3)	O^i^—As—O^viii^	109.31 (5)
O—Rb—O^v^	133.06 (3)	O^i^—As—O^ix^	109.31 (5)
O—Rb—O^vi^	67.05 (3)	O^viii^—As—O^ix^	109.80 (5)
O—Rb—O^vii^	80.84 (3)		

Symmetry codes: (i) −*x*, −*y*, *z*; (ii) *y*, −*x*, −*z*+1; (iii) −*y*, *x*, −*z*+1; (iv) −*x*+1/2, *y*, −*z*+3/4; (v) *x*−1/2, −*y*, −*z*+3/4; (vi) −*y*, −*x*+1/2, *z*+1/4; (vii) *y*, *x*−1/2, *z*+1/4; (viii) *y*, −*x*, −*z*; (ix) −*y*, *x*, −*z*.

### Hydrogen-bond geometry (Å, º)

**Table:**

*D*—H···*A*	*D*—H	H···*A*	*D*···*A*	*D*—H···*A*
O—H···O^x^	0.850 (8)	1.665 (6)	2.5125 (13)	175 (6)

Symmetry code: (x) *x*, −*y*+1/2, −*z*+1/4.
